# (4*S*,5*S*)-2-(4-Chloro­phen­yl)-1,3-dioxolane-4,5-dicarboxamide

**DOI:** 10.1107/S1600536809027494

**Published:** 2009-07-22

**Authors:** De-Cai Wang, Jing Bai, Wei Xu, Tao Gai, Hua-Quan Liu

**Affiliations:** a School of Pharmaceutical Sciences, Nanjing University of Technology, Xinmofan Road No.5 Nanjing, Nanjing 210009, People’s Republic of China

## Abstract

The title compound, C_11_H_11_ClN_2_O_4_, is an important inter­mediate for the preparation of platinum anti­cancer drugs. The dioxolane ring adopts a twist conformation with an equatorially attached chloro­phenyl substituent. In the crystal structure, mol­ecules are linked into a two-dimensional network parallel to (001) by N—H⋯O and C—H⋯O hydrogen bonds.

## Related literature

For bond-length data, see: Allen *et al.* (1987 ▶). For general background to platinum anti­cancer drugs, see: Kim *et al.* (1994 ▶); Pandey *et al.* (1997 ▶).
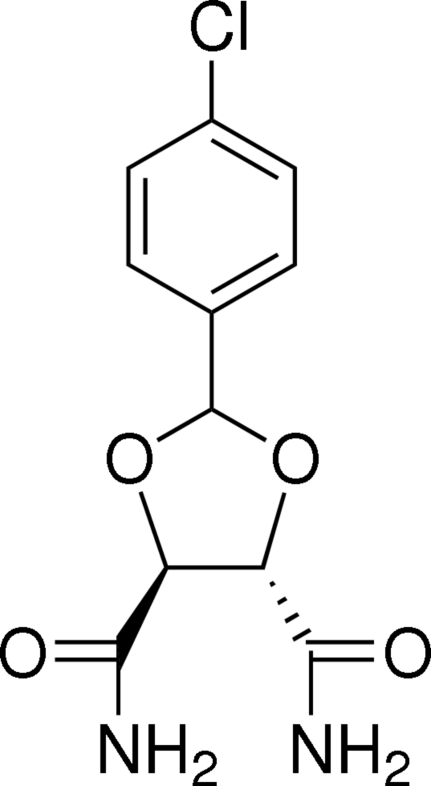


## Experimental

### 

#### Crystal data

C_11_H_11_ClN_2_O_4_
*M* *_r_* = 270.67
Monoclinic, 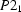

*a* = 9.2780 (19) Å
*b* = 4.7600 (10) Å
*c* = 13.245 (3) Å
β = 93.15 (3)°
*V* = 584.1 (2) Å^3^
*Z* = 2
Mo *K*α radiation
μ = 0.34 mm^−1^
*T* = 293 K
0.20 × 0.20 × 0.10 mm

#### Data collection

Enraf–Nonius CAD-4 diffractometer
Absorption correction: ψ scan (North *et al.*, 1968 ▶) *T*_min_ = 0.936, *T*_max_ = 0.967
2248 measured reflections
2113 independent reflections
1694 reflections with *I* > 2σ(*I*)
*R*_int_ = 0.027
3 standard reflections every 200 reflections intensity decay: 1%

#### Refinement

*R*[*F* ^2^ > 2σ(*F* ^2^)] = 0.048
*wR*(*F* ^2^) = 0.122
*S* = 1.07
2113 reflections
179 parameters
1 restraint
H atoms treated by a mixture of independent and constrained refinement
Δρ_max_ = 0.20 e Å^−3^
Δρ_min_ = −0.27 e Å^−3^
Absolute structure: Flack (1983 ▶), 916 Friedel pairs
Flack parameter: 0.07 (14)

### 

Data collection: *CAD-4 EXPRESS* (Enraf–Nonius, 1989 ▶); cell refinement: *CAD-4 EXPRESS*; data reduction: *XCAD4* (Harms & Wocadlo, 1995 ▶); program(s) used to solve structure: *SHELXS97* (Sheldrick, 2008 ▶); program(s) used to refine structure: *SHELXL97* (Sheldrick, 2008 ▶); molecular graphics: *SHELXTL* (Sheldrick, 2008 ▶); software used to prepare material for publication: *SHELXL97*.

## Supplementary Material

Crystal structure: contains datablocks global, I. DOI: 10.1107/S1600536809027494/ci2828sup1.cif

Structure factors: contains datablocks I. DOI: 10.1107/S1600536809027494/ci2828Isup2.hkl

Additional supplementary materials:  crystallographic information; 3D view; checkCIF report

## Figures and Tables

**Table 1:** Hydrogen-bond geometry (Å, °)

*D*—H⋯*A*	*D*—H	H⋯*A*	*D*⋯*A*	*D*—H⋯*A*
N1—H1*A*⋯O3^i^	0.79 (4)	2.20 (4)	2.979 (5)	166 (4)
N2—H2*A*⋯O3^ii^	0.87 (4)	2.42 (3)	3.173 (5)	145 (3)
N2—H2*B*⋯O4^iii^	0.81 (6)	2.26 (6)	3.012 (4)	155 (4)
C9—H9⋯O4^iii^	0.98	2.31	3.075 (4)	135

Symmetry codes: (i) 
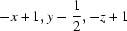
; (ii) 
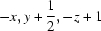
; (iii) 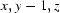.

**Fig. 1.**
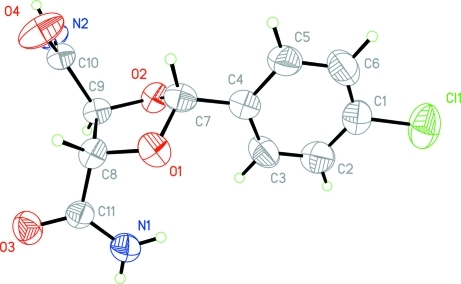
The molecular structure of the title compound, showing the atom-numbering scheme and displacement ellipsoids at the 30% probability level.

**Fig. 2.**
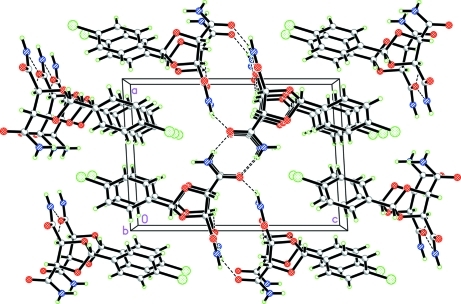
A packing diagram of the title compound. Hydrogen bonds are shown as dashed lines.

## supplementary crystallographic information

### Comment

Platinum antitumor drug is one kind of the most effective anticancer agents currently available. (2*S*,3S)-Diethyl 2,3-*O*-alkyltartrate analogues are starting materials for the syntheses of platinum complexes with antitumor activity (Kim *et al.*,1994), and are also important intermediates in organic syntheses (Pandey *et al.*, 1997). As part of our studies on the syntheses and characterizations of these compounds, we have synthesized the title compound and reported herein its crystal structure.

In the molecule of the title compound (Fig 1), the bond lengths (Allen *et al.*, 1987) and angles are within normal ranges. The dioxolane ring adopts a twist conformation and the chlorophenyl unit is equatorially attached.

In the crystal structure, N—H···O and C—H···O intermolecular hydrogen bonds (Table 1) link the molecules to form a two-dimensional network (Fig. 2) parallel to the (001).

### Experimental

4-Chlorobenzaldehyde (278 mg, 1.98 mmol), (2*S*,3S)-diethyltartrate (378 mg,1.84 mmol) and cyclohexane (10 ml) were placed in a round-bottomed flask, and 4-methylbenzenesulfonic acid (30 mg) was added. The flask was fitted with a water-distributor. The mixture was heated under reflux for 3 h. The reaction mixture was cooled to room temperature, and then transfered into a separatory funnel, washed with water (200 ml) and extracted with acetate (200 ml). The organic phase was distilled under pressure, and the residual was dissolved in anhydrous ethanol (50 ml). Then, a current of dry ammonia was passed through the reaction mixture at room temperature for about 4 h. The reaction mixture was then added dropwise to a vigorously stirred water (600 ml). The resulting colourless precipitate was obtained by filtration and dried *in vacuo* (Kim *et al.*, 1994). Single crystals suitable for X-ray analysis were obtained by slow evaporation of a methanol solution after two weeks.

### Refinement

H atoms were positioned geometrically (C—H = 0.93–0.97 Å) and included in the refinement in riding motion approximation, with *U*_iso_(H) = 1.2 or 1.5*U*_eq_ of the carrier atom.

### Crystal data

**Table:**

C_11_H_11_ClN_2_O_4_	*F*(000) = 280
*M**_r_* = 270.67	*D*_x_ = 1.539 Mg m^−^^3^
Monoclinic, *P*2_1_	Mo *K*α radiation, λ = 0.71073 Å
Hall symbol: P 2yb	Cell parameters from 25 reflections
*a* = 9.2780 (19) Å	θ = 9–13°
*b* = 4.760 (1) Å	µ = 0.34 mm^−^^1^
*c* = 13.245 (3) Å	*T* = 293 K
β = 93.15 (3)°	Block, colourless
*V* = 584.1 (2) Å^3^	0.20 × 0.20 × 0.10 mm
*Z* = 2	

### Data collection

**Table:**

Enraf–Nonius CAD-4 diffractometer	1694 reflections with *I* > 2σ(*I*)
Radiation source: fine-focus sealed tube	*R*_int_ = 0.027
graphite	θ_max_ = 25.3°, θ_min_ = 1.5°
ω/2θ scans	*h* = 0→11
Absorption correction: ψ scan (North *et al.*, 1968)	*k* = −5→5
*T*_min_ = 0.936, *T*_max_ = 0.967	*l* = −15→15
2248 measured reflections	3 standard reflections every 200 reflections
2113 independent reflections	intensity decay: 1%

### Refinement

**Table:**

Refinement on *F*^2^	Secondary atom site location: difference Fourier map
Least-squares matrix: full	Hydrogen site location: inferred from neighbouring sites
*R*[*F*^2^ > 2σ(*F*^2^)] = 0.048	H atoms treated by a mixture of independent and constrained refinement
*wR*(*F*^2^) = 0.122	*w* = 1/[σ^2^(*F*_o_^2^) + (0.0601*P*)^2^ + 0.0558*P*] where *P* = (*F*_o_^2^ + 2*F*_c_^2^)/3
*S* = 1.07	(Δ/σ)_max_ = 0.001
2113 reflections	Δρ_max_ = 0.20 e Å^−^^3^
179 parameters	Δρ_min_ = −0.26 e Å^−^^3^
1 restraint	Absolute structure: Flack (1983), 916 Friedel pairs
Primary atom site location: structure-invariant direct methods	Flack parameter: 0.07 (14)

### Special details

**Table:**

Geometry. All e.s.d.'s (except the e.s.d. in the dihedral angle between two l.s. planes) are estimated using the full covariance matrix. The cell e.s.d.'s are taken into account individually in the estimation of e.s.d.'s in distances, angles and torsion angles; correlations between e.s.d.'s in cell parameters are only used when they are defined by crystal symmetry. An approximate (isotropic) treatment of cell e.s.d.'s is used for estimating e.s.d.'s involving l.s. planes.
Refinement. Refinement of *F*^2^ against ALL reflections. The weighted *R*-factor *wR* and goodness of fit *S* are based on *F*^2^, conventional *R*-factors *R* are based on *F*, with *F* set to zero for negative *F*^2^. The threshold expression of *F*^2^ > σ(*F*^2^) is used only for calculating *R*-factors(gt) *etc*. and is not relevant to the choice of reflections for refinement. *R*-factors based on *F*^2^ are statistically about twice as large as those based on *F*, and *R*- factors based on ALL data will be even larger.

### Fractional atomic coordinates and isotropic or equivalent isotropic displacement parameters (Å^2^)

**Table:**

	*x*	*y*	*z*	*U*_iso_*/*U*_eq_	
Cl1	0.35368 (14)	−0.0236 (4)	−0.21514 (8)	0.0925 (5)	
O1	0.2854 (2)	0.2859 (5)	0.26692 (18)	0.0475 (6)	
O2	0.0917 (2)	0.0071 (5)	0.24181 (16)	0.0461 (6)	
O3	0.3515 (3)	0.0218 (6)	0.51614 (17)	0.0568 (7)	
O4	−0.0323 (3)	0.4727 (5)	0.3827 (2)	0.0657 (8)	
N1	0.4522 (4)	−0.0804 (9)	0.3700 (3)	0.0597 (10)	
H1A	0.516 (4)	−0.168 (8)	0.397 (3)	0.038 (10)*	
H1B	0.451 (4)	−0.069 (10)	0.305 (3)	0.064 (13)*	
N2	−0.1472 (4)	0.0634 (7)	0.3925 (3)	0.0458 (7)	
H2A	−0.230 (4)	0.139 (7)	0.403 (2)	0.034 (9)*	
H2B	−0.140 (5)	−0.105 (12)	0.399 (3)	0.071 (15)*	
C1	0.2948 (4)	0.0470 (10)	−0.0955 (3)	0.0573 (10)	
C2	0.3508 (5)	−0.1037 (10)	−0.0134 (3)	0.0675 (12)	
H2	0.4179	−0.2455	−0.0224	0.081*	
C3	0.3072 (4)	−0.0438 (10)	0.0813 (3)	0.0618 (10)	
H3	0.3440	−0.1476	0.1363	0.074*	
C4	0.2085 (4)	0.1701 (7)	0.0964 (3)	0.0467 (9)	
C5	0.1527 (4)	0.3126 (9)	0.0133 (3)	0.0614 (11)	
H5	0.0852	0.4541	0.0216	0.074*	
C6	0.1949 (5)	0.2500 (11)	−0.0830 (3)	0.0701 (12)	
H6	0.1549	0.3468	−0.1387	0.084*	
C7	0.1632 (4)	0.2384 (7)	0.1998 (3)	0.0471 (8)	
H7	0.1002	0.4038	0.1976	0.057*	
C8	0.2436 (3)	0.2201 (7)	0.3666 (2)	0.0392 (7)	
H8	0.2264	0.3935	0.4039	0.047*	
C9	0.1005 (3)	0.0548 (7)	0.3490 (2)	0.0378 (7)	
H9	0.1064	−0.1248	0.3852	0.045*	
C10	−0.0327 (3)	0.2166 (7)	0.3777 (3)	0.0395 (8)	
C11	0.3560 (3)	0.0449 (8)	0.4236 (2)	0.0423 (8)	

### Atomic displacement parameters (Å^2^)

**Table:**

	*U*^11^	*U*^22^	*U*^33^	*U*^12^	*U*^13^	*U*^23^
Cl1	0.1056 (9)	0.1139 (10)	0.0597 (6)	−0.0231 (10)	0.0197 (6)	−0.0196 (7)
O1	0.0522 (13)	0.0425 (14)	0.0473 (13)	−0.0157 (11)	−0.0015 (11)	0.0055 (11)
O2	0.0474 (12)	0.0375 (13)	0.0539 (13)	−0.0122 (12)	0.0066 (10)	−0.0116 (12)
O3	0.0565 (14)	0.0719 (19)	0.0423 (13)	−0.0098 (15)	0.0051 (11)	0.0030 (14)
O4	0.0582 (15)	0.0206 (12)	0.120 (2)	0.0031 (12)	0.0207 (14)	−0.0055 (15)
N1	0.055 (2)	0.074 (3)	0.050 (2)	0.0199 (19)	−0.0040 (17)	−0.0013 (18)
N2	0.0402 (17)	0.0289 (17)	0.069 (2)	0.0016 (14)	0.0126 (14)	0.0046 (14)
C1	0.057 (2)	0.064 (3)	0.051 (2)	−0.019 (2)	0.0029 (17)	−0.008 (2)
C2	0.071 (3)	0.065 (3)	0.066 (3)	0.011 (2)	−0.003 (2)	−0.019 (2)
C3	0.078 (3)	0.058 (2)	0.048 (2)	0.012 (2)	−0.0093 (18)	−0.003 (2)
C4	0.049 (2)	0.0377 (19)	0.053 (2)	−0.0036 (16)	−0.0039 (17)	−0.0016 (16)
C5	0.057 (2)	0.061 (3)	0.066 (3)	0.009 (2)	0.0015 (19)	0.012 (2)
C6	0.073 (3)	0.082 (3)	0.055 (2)	−0.005 (3)	−0.006 (2)	0.016 (2)
C7	0.049 (2)	0.0352 (18)	0.057 (2)	0.0005 (17)	−0.0044 (17)	0.0031 (16)
C8	0.0428 (18)	0.0303 (17)	0.0450 (18)	−0.0061 (15)	0.0073 (15)	−0.0031 (14)
C9	0.0388 (16)	0.0245 (15)	0.0500 (19)	−0.0048 (14)	0.0021 (14)	−0.0002 (13)
C10	0.0395 (17)	0.0312 (19)	0.0479 (19)	−0.0016 (15)	0.0023 (15)	0.0005 (15)
C11	0.0343 (16)	0.045 (2)	0.0475 (19)	−0.0100 (16)	0.0032 (14)	−0.0074 (17)

### Geometric parameters (Å, °)

**Table:**

Cl1—C1	1.738 (4)	C2—C3	1.370 (5)
O1—C7	1.420 (4)	C2—H2	0.93
O1—C8	1.431 (4)	C3—C4	1.391 (5)
O2—C7	1.415 (4)	C3—H3	0.93
O2—C9	1.436 (4)	C4—C5	1.370 (5)
O3—C11	1.233 (4)	C4—C7	1.490 (5)
O4—C10	1.221 (4)	C5—C6	1.387 (6)
N1—C11	1.313 (5)	C5—H5	0.93
N1—H1A	0.79 (4)	C6—H6	0.93
N1—H1B	0.86 (4)	C7—H7	0.98
N2—C10	1.312 (5)	C8—C11	1.506 (5)
N2—H2A	0.86 (3)	C8—C9	1.550 (4)
N2—H2B	0.81 (6)	C8—H8	0.98
C1—C6	1.355 (6)	C9—C10	1.522 (4)
C1—C2	1.379 (6)	C9—H9	0.98
			
C7—O1—C8	107.2 (2)	C5—C6—H6	120.2
C7—O2—C9	105.3 (2)	O2—C7—O1	104.7 (3)
C11—N1—H1A	120 (3)	O2—C7—C4	110.7 (3)
C11—N1—H1B	123 (3)	O1—C7—C4	110.8 (3)
H1A—N1—H1B	117 (4)	O2—C7—H7	110.2
C10—N2—H2A	122 (2)	O1—C7—H7	110.2
C10—N2—H2B	121 (3)	C4—C7—H7	110.2
H2A—N2—H2B	117 (4)	O1—C8—C11	111.6 (3)
C6—C1—C2	120.4 (4)	O1—C8—C9	104.2 (2)
C6—C1—Cl1	120.0 (3)	C11—C8—C9	111.0 (3)
C2—C1—Cl1	119.6 (3)	O1—C8—H8	109.9
C3—C2—C1	119.7 (4)	C11—C8—H8	109.9
C3—C2—H2	120.1	C9—C8—H8	109.9
C1—C2—H2	120.1	O2—C9—C10	108.9 (2)
C2—C3—C4	121.0 (4)	O2—C9—C8	103.3 (2)
C2—C3—H3	119.5	C10—C9—C8	114.0 (3)
C4—C3—H3	119.5	O2—C9—H9	110.1
C5—C4—C3	118.0 (4)	C10—C9—H9	110.1
C5—C4—C7	121.2 (3)	C8—C9—H9	110.1
C3—C4—C7	120.9 (3)	O4—C10—N2	123.2 (3)
C4—C5—C6	121.3 (4)	O4—C10—C9	121.2 (3)
C4—C5—H5	119.4	N2—C10—C9	115.5 (3)
C6—C5—H5	119.4	O3—C11—N1	123.9 (3)
C1—C6—C5	119.7 (4)	O3—C11—C8	119.2 (3)
C1—C6—H6	120.2	N1—C11—C8	116.9 (3)
			
C6—C1—C2—C3	−1.3 (6)	C3—C4—C7—O1	52.9 (5)
Cl1—C1—C2—C3	178.4 (3)	C7—O1—C8—C11	134.7 (3)
C1—C2—C3—C4	−0.9 (7)	C7—O1—C8—C9	14.8 (3)
C2—C3—C4—C5	2.1 (6)	C7—O2—C9—C10	91.5 (3)
C2—C3—C4—C7	−178.9 (4)	C7—O2—C9—C8	−30.0 (3)
C3—C4—C5—C6	−1.2 (6)	O1—C8—C9—O2	9.3 (3)
C7—C4—C5—C6	179.8 (4)	C11—C8—C9—O2	−111.0 (3)
C2—C1—C6—C5	2.2 (6)	O1—C8—C9—C10	−108.7 (3)
Cl1—C1—C6—C5	−177.5 (3)	C11—C8—C9—C10	130.9 (3)
C4—C5—C6—C1	−1.0 (7)	O2—C9—C10—O4	−93.1 (4)
C9—O2—C7—O1	40.4 (3)	C8—C9—C10—O4	21.7 (5)
C9—O2—C7—C4	159.7 (3)	O2—C9—C10—N2	84.4 (4)
C8—O1—C7—O2	−34.2 (3)	C8—C9—C10—N2	−160.8 (3)
C8—O1—C7—C4	−153.5 (3)	O1—C8—C11—O3	164.5 (3)
C5—C4—C7—O2	116.2 (4)	C9—C8—C11—O3	−79.7 (4)
C3—C4—C7—O2	−62.8 (4)	O1—C8—C11—N1	−17.0 (4)
C5—C4—C7—O1	−128.1 (4)	C9—C8—C11—N1	98.8 (4)

### Hydrogen-bond geometry (Å, °)

**Table:**

*D*—H···*A*	*D*—H	H···*A*	*D*···*A*	*D*—H···*A*
N1—H1A···O3^i^	0.79 (4)	2.20 (4)	2.979 (5)	166 (4)
N2—H2A···O3^ii^	0.87 (4)	2.42 (3)	3.173 (5)	145 (3)
N2—H2B···O4^iii^	0.81 (6)	2.26 (6)	3.012 (4)	155 (4)
C9—H9···O4^iii^	0.98	2.31	3.075 (4)	135

Symmetry codes: (i) −*x*+1, *y*−1/2, −*z*+1; (ii) −*x*, *y*+1/2, −*z*+1; (iii) *x*, *y*−1, *z*.
